# 2-Chloro­benzohydrazide

**DOI:** 10.1107/S1600536812012640

**Published:** 2012-03-31

**Authors:** Shakeel Ahmad, Abdul Jabbar, Muhammad Tahir Hussain, M. Nawaz Tahir

**Affiliations:** a Department of Chemistry, G.C. University, Faisalabad 38000, Pakistan; b Department of Applied Sciences, National Textile University, Faisalabad 37610, Pakistan; c University of Sargodha, Department of Physics, Sargodha, Pakistan

## Abstract

The asymmetric unit of the the title compound, C_7_H_7_ClN_2_O, contains two mol­ecules in which the chloro­phenyl and the formic hydrazide units are almost planar (r.m.s. deviations of 0.0081 and 0.0100 Å, respectively, in one mol­ecule and 0.0069 and 0.0150 Å in the other) and are oriented with respect to each other at dihedral angles of 56.8 (2) and 56.9 (2)°. In the crystal, the mol­ecules are consolidated in the form of polymeric chains extending along [010]. *R*_3_^3^(10) ring motifs exist due to N—H⋯O and N—H⋯N hydrogen bonds.

## Related literature

For a related structure, see: Zareef *et al.* (2006 ▶). For graph-set notation, see: Bernstein *et al.* (1995 ▶). For the synthetic method, see: Moise *et al.* (2009 ▶).
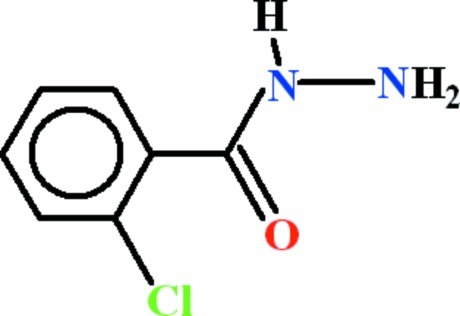


## Experimental

### 

#### Crystal data

C_7_H_7_ClN_2_O
*M* *_r_* = 170.60
Monoclinic, 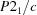

*a* = 25.7589 (16) Å
*b* = 4.9618 (3) Å
*c* = 12.9205 (8) Å
β = 103.648 (3)°
*V* = 1604.75 (17) Å^3^
*Z* = 8
Mo *K*α radiation
μ = 0.42 mm^−1^
*T* = 296 K
0.34 × 0.14 × 0.12 mm

#### Data collection

Bruker Kappa APEXII CCD diffractometer
Absorption correction: multi-scan (*SADABS*; Bruker, 2009 ▶) *T*_min_ = 0.979, *T*_max_ = 0.988
11488 measured reflections
3091 independent reflections
2089 reflections with *I* > 2σ(*I*)
*R*_int_ = 0.039

#### Refinement

*R*[*F* ^2^ > 2σ(*F* ^2^)] = 0.083
*wR*(*F* ^2^) = 0.217
*S* = 1.08
3091 reflections
211 parameters
H atoms treated by a mixture of independent and constrained refinement
Δρ_max_ = 0.80 e Å^−3^
Δρ_min_ = −0.47 e Å^−3^

### 

Data collection: *APEX2* (Bruker, 2009 ▶); cell refinement: *SAINT* (Bruker, 2009 ▶); data reduction: *SAINT*; program(s) used to solve structure: *SHELXS97* (Sheldrick, 2008 ▶); program(s) used to refine structure: *SHELXL97* (Sheldrick, 2008 ▶); molecular graphics: *ORTEP-3 for Windows* (Farrugia, 1997 ▶) and *PLATON* (Spek, 2009 ▶); software used to prepare material for publication: *WinGX* (Farrugia, 1999 ▶) and *PLATON*.

## Supplementary Material

Crystal structure: contains datablock(s) global, I. DOI: 10.1107/S1600536812012640/ez2284sup1.cif

Structure factors: contains datablock(s) I. DOI: 10.1107/S1600536812012640/ez2284Isup2.hkl

Supplementary material file. DOI: 10.1107/S1600536812012640/ez2284Isup3.cml

Additional supplementary materials:  crystallographic information; 3D view; checkCIF report

## Figures and Tables

**Table 1:** Hydrogen-bond geometry (Å, °)

*D*—H⋯*A*	*D*—H	H⋯*A*	*D*⋯*A*	*D*—H⋯*A*
N1—H1⋯O1^i^	0.86	2.05	2.825 (5)	149
N2—H2*A*⋯N2^ii^	0.89 (6)	2.27 (6)	3.151 (7)	172 (5)
N3—H3*A*⋯O2^iii^	0.86	2.10	2.812 (5)	140
N4—H4*B*⋯N4^iv^	0.80 (6)	2.40 (6)	3.155 (6)	158 (5)

Symmetry codes: (i) 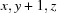; (ii) 
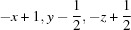
; (iii) 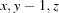; (iv) 
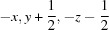
.

**Fig. 1.**
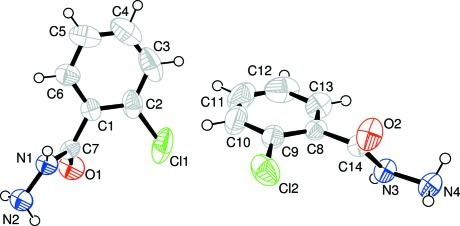
View of the title compound with the atom numbering scheme. The displacement ellipsoids are drawn at the 50% probability level. H-atoms are shown as small circles of arbitrary radii.

**Fig. 2.**
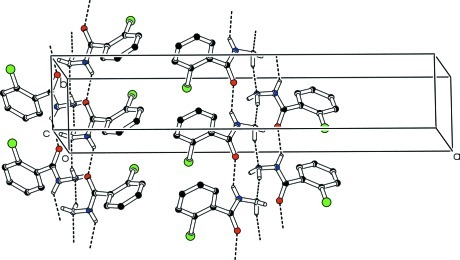
Partial packing diagram (PLATON; Spek, 2009) which shows that the molecules form polymeric chains extending along the [0 1 0] direction forming R33(10) ring motifs.

## supplementary crystallographic information

### Comment

The title compound (I), (Fig. 1) has been synthesized as a precursor for the preparation of various substituted triazole derivatives.

We have reported the crystal structures of *N*-2-bromobenzoylhydrazide (Zareef *et al.*, 2006), which is related to (I).

In (I), two molecules are present in the asymmetric unit, which differ slightly from each other geometrically. In one molecule, the chlorophenyl group A (C1–C6/Cl1) and the formic hydrazide moiety B (O1/C7/N1/N2) are planar with r. m. s. deviations of 0.0081Å and 0.0100 Å, respectively. The dihedral angle between A/B is 56.8 (2)°. In second molecule, the chlorophenyl group C (C8–C13/Cl2) and the formic hydrazide moiety D (O2/C14/N3/N4) are also planar with r. m. s. deviation of 0.0069Å and 0.0150 Å, respectively and the dihedral angle between C/D is 56.89 (20)°. Each molecule is connected to symmetry related neighbors through classical intermolecular H–bonding of the N—H···O or N—H···N type (Table 1, Fig. 2) with *R*_3_^3^(10) ring motifs (Bernstein *et al.*, 1995) to generate one-dimensional polymeric chains along the [0 1 0] direction.

### Experimental

2-Chlorobenzoic acid (3.44 g, 22 mmol) was converted to methyl 2-chlorobenzoate by refluxing in methanol (20 ml) in the presence of a catalytic amount of sulfuric acid. This ester was converted into the title compound, 2-chlorobenzoylhydrazide, by refluxing with hydrazine hydrate (80 %, 10 ml) in dry methanol using the literature procedure (Moise *et al.*, 2009). M.p. 379-380K.

### Refinement

The coordinates of the H-atoms of the NH_2_ groups were refined. The remaining H atoms were positioned geometrically with (N–H = 0.86 and C–H = 0.93 Å) and refined as riding with *U*_iso_(H) = *xU*_eq_(C, N), where *x* = 1.2 for all H-atoms.

### Crystal data

**Table:**

C_7_H_7_ClN_2_O	*F*(000) = 704
*M**_r_* = 170.60	*D*_x_ = 1.412 Mg m^−^^3^
Monoclinic, *P*2_1_/*c*	Mo *K*α radiation, λ = 0.71073 Å
Hall symbol: -P 2ybc	Cell parameters from 2089 reflections
*a* = 25.7589 (16) Å	θ = 0.8–26.0°
*b* = 4.9618 (3) Å	µ = 0.42 mm^−^^1^
*c* = 12.9205 (8) Å	*T* = 296 K
β = 103.648 (3)°	Needle, colorless
*V* = 1604.75 (17) Å^3^	0.34 × 0.14 × 0.12 mm
*Z* = 8	

### Data collection

**Table:**

Bruker Kappa APEXII CCD diffractometer	3091 independent reflections
Radiation source: fine-focus sealed tube	2089 reflections with *I* > 2σ(*I*)
Graphite monochromator	*R*_int_ = 0.039
Detector resolution: 7.6 pixels mm^-1^	θ_max_ = 26.0°, θ_min_ = 0.8°
ω scans	*h* = −31→31
Absorption correction: multi-scan (*SADABS*; Bruker, 2009)	*k* = −6→5
*T*_min_ = 0.979, *T*_max_ = 0.988	*l* = −15→15
11488 measured reflections	

### Refinement

**Table:**

Refinement on *F*^2^	Primary atom site location: structure-invariant direct methods
Least-squares matrix: full	Secondary atom site location: difference Fourier map
*R*[*F*^2^ > 2σ(*F*^2^)] = 0.083	Hydrogen site location: inferred from neighbouring sites
*wR*(*F*^2^) = 0.217	H atoms treated by a mixture of independent and constrained refinement
*S* = 1.08	*w* = 1/[σ^2^(*F*_o_^2^) + (0.0577*P*)^2^ + 4.8436*P*] where *P* = (*F*_o_^2^ + 2*F*_c_^2^)/3
3091 reflections	(Δ/σ)_max_ < 0.001
211 parameters	Δρ_max_ = 0.80 e Å^−^^3^
0 restraints	Δρ_min_ = −0.47 e Å^−^^3^

### Special details

**Table:**

Geometry. Bond distances, angles *etc*. have been calculated using the rounded fractional coordinates. All su's are estimated from the variances of the (full) variance-covariance matrix. The cell e.s.d.'s are taken into account in the estimation of distances, angles and torsion angles
Refinement. Refinement of *F*^2^ against ALL reflections. The weighted *R*-factor *wR* and goodness of fit *S* are based on *F*^2^, conventional *R*-factors *R* are based on *F*, with *F* set to zero for negative *F*^2^. The threshold expression of *F*^2^ > σ(*F*^2^) is used only for calculating *R*-factors(gt) *etc*. and is not relevant to the choice of reflections for refinement. *R*-factors based on *F*^2^ are statistically about twice as large as those based on *F*, and *R*- factors based on ALL data will be even larger.

### Fractional atomic coordinates and isotropic or equivalent isotropic displacement parameters (Å^2^)

**Table:**

	*x*	*y*	*z*	*U*_iso_*/*U*_eq_	
Cl1	0.32498 (8)	−0.2330 (4)	0.33851 (15)	0.1011 (8)	
O1	0.45056 (16)	−0.1875 (7)	0.4080 (3)	0.0622 (15)	
N1	0.45512 (15)	0.2531 (8)	0.3704 (3)	0.0425 (12)	
N2	0.49903 (18)	0.2265 (9)	0.3247 (4)	0.0481 (16)	
C1	0.39139 (18)	0.1163 (10)	0.4673 (4)	0.0432 (17)	
C2	0.3415 (2)	0.0002 (13)	0.4426 (4)	0.067 (2)	
C3	0.3025 (3)	0.0750 (18)	0.4998 (7)	0.093 (3)	
C4	0.3167 (3)	0.256 (2)	0.5818 (7)	0.102 (4)	
C5	0.3654 (3)	0.3692 (15)	0.6064 (5)	0.080 (3)	
C6	0.4023 (2)	0.3033 (11)	0.5501 (4)	0.0570 (17)	
C7	0.43389 (18)	0.0452 (9)	0.4120 (3)	0.0397 (16)	
Cl2	0.16784 (6)	0.7856 (3)	−0.01597 (14)	0.0751 (6)	
O2	0.04466 (14)	0.6942 (7)	−0.0529 (3)	0.0583 (11)	
N3	0.04524 (14)	0.2531 (7)	−0.0886 (3)	0.0399 (12)	
N4	−0.00065 (18)	0.2615 (9)	−0.1749 (3)	0.0461 (14)	
C8	0.10837 (18)	0.4182 (10)	0.0635 (3)	0.0422 (16)	
C9	0.15626 (19)	0.5612 (12)	0.0799 (4)	0.0546 (19)	
C10	0.1956 (2)	0.5229 (16)	0.1706 (6)	0.081 (3)	
C11	0.1879 (3)	0.348 (2)	0.2458 (6)	0.094 (3)	
C12	0.1414 (3)	0.2024 (17)	0.2324 (5)	0.089 (3)	
C13	0.1015 (2)	0.2401 (12)	0.1409 (4)	0.0598 (19)	
C14	0.06346 (18)	0.4680 (9)	−0.0317 (3)	0.0388 (16)	
H1	0.44118	0.41011	0.37190	0.0512*	
H2A	0.496 (2)	0.082 (12)	0.283 (4)	0.0577*	
H2B	0.523 (2)	0.180 (13)	0.366 (4)	0.0577*	
H3	0.26820	0.00263	0.48178	0.1112*	
H4	0.29213	0.30088	0.62131	0.1223*	
H5	0.37401	0.49282	0.66193	0.0959*	
H6	0.43564	0.38561	0.56747	0.0681*	
H3A	0.06170	0.10231	−0.07305	0.0475*	
H4A	−0.026 (2)	0.298 (11)	−0.141 (4)	0.0556*	
H4B	0.006 (2)	0.363 (12)	−0.218 (4)	0.0556*	
H10	0.22764	0.61712	0.18040	0.0978*	
H11	0.21460	0.32500	0.30771	0.1128*	
H12	0.13680	0.08032	0.28412	0.1061*	
H13	0.06966	0.14431	0.13169	0.0717*	

### Atomic displacement parameters (Å^2^)

**Table:**

	*U*^11^	*U*^22^	*U*^33^	*U*^12^	*U*^13^	*U*^23^
Cl1	0.0933 (12)	0.1012 (16)	0.0900 (12)	−0.0555 (11)	−0.0160 (9)	0.0057 (11)
O1	0.083 (3)	0.0225 (19)	0.088 (3)	−0.0036 (18)	0.034 (2)	−0.0005 (19)
N1	0.055 (2)	0.021 (2)	0.055 (2)	−0.0018 (17)	0.0199 (19)	−0.0024 (18)
N2	0.057 (3)	0.032 (2)	0.060 (3)	−0.002 (2)	0.023 (2)	−0.002 (2)
C1	0.046 (3)	0.034 (3)	0.050 (3)	−0.002 (2)	0.012 (2)	0.013 (2)
C2	0.054 (3)	0.076 (5)	0.067 (3)	−0.018 (3)	0.006 (3)	0.022 (3)
C3	0.053 (3)	0.115 (7)	0.112 (6)	−0.008 (4)	0.023 (4)	0.046 (5)
C4	0.080 (5)	0.129 (8)	0.112 (6)	0.017 (5)	0.052 (5)	0.015 (6)
C5	0.095 (5)	0.082 (5)	0.073 (4)	0.020 (4)	0.042 (4)	0.005 (4)
C6	0.066 (3)	0.048 (3)	0.062 (3)	0.004 (3)	0.025 (3)	0.002 (3)
C7	0.055 (3)	0.022 (3)	0.041 (2)	−0.005 (2)	0.009 (2)	−0.003 (2)
Cl2	0.0653 (9)	0.0623 (10)	0.1057 (12)	−0.0121 (8)	0.0360 (8)	−0.0044 (9)
O2	0.067 (2)	0.0225 (19)	0.075 (2)	0.0001 (16)	−0.0040 (18)	0.0014 (17)
N3	0.049 (2)	0.019 (2)	0.048 (2)	0.0053 (16)	0.0041 (16)	−0.0001 (17)
N4	0.056 (2)	0.034 (3)	0.044 (2)	−0.001 (2)	0.0032 (19)	−0.0001 (19)
C8	0.046 (3)	0.037 (3)	0.042 (2)	0.008 (2)	0.007 (2)	−0.006 (2)
C9	0.046 (3)	0.055 (4)	0.060 (3)	0.009 (2)	0.007 (2)	−0.019 (3)
C10	0.055 (3)	0.094 (6)	0.084 (5)	0.005 (4)	−0.006 (3)	−0.032 (4)
C11	0.079 (5)	0.126 (7)	0.061 (4)	0.036 (5)	−0.017 (4)	−0.020 (5)
C12	0.112 (6)	0.096 (6)	0.056 (4)	0.051 (5)	0.017 (4)	0.020 (4)
C13	0.070 (3)	0.051 (4)	0.056 (3)	0.007 (3)	0.010 (3)	0.010 (3)
C14	0.049 (3)	0.024 (3)	0.044 (2)	−0.002 (2)	0.012 (2)	0.002 (2)

### Geometric parameters (Å, º)

**Table:**

Cl1—C2	1.749 (6)	C3—C4	1.371 (13)
Cl2—C9	1.743 (6)	C4—C5	1.342 (11)
O1—C7	1.237 (6)	C5—C6	1.366 (9)
O2—C14	1.228 (6)	C3—H3	0.9300
N1—N2	1.400 (6)	C4—H4	0.9300
N1—C7	1.338 (6)	C5—H5	0.9300
N1—H1	0.8600	C6—H6	0.9300
N2—H2A	0.89 (6)	C8—C14	1.497 (6)
N2—H2B	0.75 (5)	C8—C9	1.395 (7)
N3—C14	1.317 (6)	C8—C13	1.377 (7)
N3—N4	1.421 (6)	C9—C10	1.370 (9)
N3—H3A	0.8600	C10—C11	1.352 (11)
N4—H4A	0.89 (5)	C11—C12	1.374 (12)
N4—H4B	0.80 (6)	C12—C13	1.384 (9)
C1—C2	1.375 (7)	C10—H10	0.9300
C1—C7	1.484 (7)	C11—H11	0.9300
C1—C6	1.394 (7)	C12—H12	0.9300
C2—C3	1.430 (10)	C13—H13	0.9300
			
N2—N1—C7	123.0 (4)	C5—C4—H4	119.00
C7—N1—H1	118.00	C3—C4—H4	119.00
N2—N1—H1	119.00	C6—C5—H5	120.00
N1—N2—H2B	110 (4)	C4—C5—H5	120.00
H2A—N2—H2B	97 (6)	C1—C6—H6	119.00
N1—N2—H2A	112 (3)	C5—C6—H6	119.00
N4—N3—C14	122.3 (4)	C9—C8—C13	118.5 (4)
C14—N3—H3A	119.00	C13—C8—C14	119.7 (4)
N4—N3—H3A	119.00	C9—C8—C14	121.7 (4)
N3—N4—H4B	107 (4)	Cl2—C9—C10	118.7 (4)
H4A—N4—H4B	121 (5)	C8—C9—C10	120.6 (5)
N3—N4—H4A	101 (3)	Cl2—C9—C8	120.7 (4)
C6—C1—C7	119.3 (4)	C9—C10—C11	120.0 (6)
C2—C1—C7	122.9 (5)	C10—C11—C12	121.2 (7)
C2—C1—C6	117.7 (5)	C11—C12—C13	119.1 (7)
Cl1—C2—C3	119.8 (5)	C8—C13—C12	120.7 (5)
Cl1—C2—C1	120.0 (4)	O2—C14—C8	121.5 (4)
C1—C2—C3	120.2 (6)	N3—C14—C8	115.4 (4)
C2—C3—C4	118.5 (7)	O2—C14—N3	123.1 (4)
C3—C4—C5	121.4 (8)	C9—C10—H10	120.00
C4—C5—C6	120.2 (7)	C11—C10—H10	120.00
C1—C6—C5	121.9 (5)	C10—C11—H11	120.00
O1—C7—C1	123.0 (4)	C12—C11—H11	119.00
N1—C7—C1	115.3 (4)	C11—C12—H12	120.00
O1—C7—N1	121.6 (4)	C13—C12—H12	120.00
C4—C3—H3	121.00	C8—C13—H13	120.00
C2—C3—H3	121.00	C12—C13—H13	120.00
			
N2—N1—C7—O1	−3.4 (7)	C3—C4—C5—C6	−0.6 (13)
N2—N1—C7—C1	173.6 (4)	C4—C5—C6—C1	−0.9 (10)
N4—N3—C14—O2	−5.1 (7)	C13—C8—C9—Cl2	179.0 (4)
N4—N3—C14—C8	173.5 (4)	C13—C8—C9—C10	0.6 (8)
C7—C1—C2—Cl1	−2.5 (7)	C14—C8—C9—Cl2	−5.3 (7)
C7—C1—C2—C3	179.2 (6)	C14—C8—C9—C10	176.3 (5)
C2—C1—C6—C5	0.7 (8)	C9—C8—C13—C12	−0.5 (8)
C7—C1—C6—C5	−177.6 (5)	C14—C8—C13—C12	−176.3 (5)
C2—C1—C7—O1	−57.1 (7)	C9—C8—C14—O2	−54.9 (7)
C2—C1—C7—N1	125.9 (5)	C9—C8—C14—N3	126.5 (5)
C6—C1—C7—O1	121.1 (5)	C13—C8—C14—O2	120.7 (5)
C6—C1—C7—N1	−55.9 (6)	C13—C8—C14—N3	−57.9 (6)
C6—C1—C2—C3	1.0 (9)	Cl2—C9—C10—C11	−179.3 (6)
C6—C1—C2—Cl1	179.2 (4)	C8—C9—C10—C11	−0.9 (10)
C1—C2—C3—C4	−2.5 (11)	C9—C10—C11—C12	1.2 (12)
Cl1—C2—C3—C4	179.3 (7)	C10—C11—C12—C13	−1.2 (12)
C2—C3—C4—C5	2.3 (13)	C11—C12—C13—C8	0.8 (10)

### Hydrogen-bond geometry (Å, º)

**Table:**

*D*—H···*A*	*D*—H	H···*A*	*D*···*A*	*D*—H···*A*
N1—H1···O1^i^	0.86	2.05	2.825 (5)	149
N2—H2*A*···N2^ii^	0.89 (6)	2.27 (6)	3.151 (7)	172 (5)
N3—H3*A*···O2^iii^	0.86	2.10	2.812 (5)	140
N4—H4*B*···N4^iv^	0.80 (6)	2.40 (6)	3.155 (6)	158 (5)

Symmetry codes: (i) *x*, *y*+1, *z*; (ii) −*x*+1, *y*−1/2, −*z*+1/2; (iii) *x*, *y*−1, *z*; (iv) −*x*, *y*+1/2, −*z*−1/2.
